# The Contribution of Y Chromosome Genes to Spontaneous Differentiation of Human Embryonic Stem Cells into Embryoid Bodies In Vitro

**DOI:** 10.22074/cellj.2021.7145

**Published:** 2021-03-01

**Authors:** Simin Nafian Dehkordi, Farzaneh Khani, Seyedeh Nafiseh Hassani, Hossein Baharvand, Hamid Reza Soleimanpour-lichaei, Ghasem Hosseini Salekdeh

**Affiliations:** 1. Department of Stem Cells and Regenerative Medicine, Institute of Medical Biotechnology, National Institute of Genetic Engineering and Biotechnology (NIGEB), Tehran, Iran; 2. Department of Molecular Systems Biology, Cell Science Research Center, Royan Institute for Stem Cell Biology and Technology, ACECR, Tehran, Iran; 3. Department of Stem Cells and Developmental Biology, Cell Science Research Center, Royan Institute for Stem Cell Biology and Technology, ACECR, Tehran, Iran; 4. Department of Developmental Biology, University of Science and Culture, Tehran, Iran; 5. Department of Systems Biology, Agricultural Biotechnology Research Institute of Iran, Karaj, Iran; 6. Department of Molecular Sciences, Macquarie University, Sydney, NSW, Australia

**Keywords:** Embryoid Bodies, Human Embryonic Stem Cells, Human Y Chromosome Proteome Project, RH6 cell line, Spontaneous Differentiation

## Abstract

**Objective:**

Sexual dimorphism in mammals can be described as subsequent transcriptional differences from their distinct sex chromosome complements. Following X inactivation in females, the Y chromosome is the major genetic difference between sexes. In this study, we used a male embryonic stem cell line (Royan H6) to identify the potential role of the male-specific region of the Y chromosome (MSY) during spontaneous differentiation into embryoid bodies (EBs) as a model of early embryonic development.

**Materials and Methods:**

In this experimental study, RH6 cells were cultured on inactivated feeder layers and Matrigel. In a dynamic suspension system, aggregates were generated in the same size and were spontaneously differentiated into EBs. During differentiation, expression patterns of specific markers for three germ layers were compared with MSY genes.

**Results:**

Spontaneous differentiation was determined by downregulation of pluripotent markers and upregulation of fourteen differentiation markers. Upregulation of the ectoderm markers was observed on days 4 and 16, whereas mesoderm markers were upregulated on the 8th day and endodermic markers on days 12-16. Mesoderm markers correlated with 8 MSY genes namely *DDX3Y, RPS4Y1, KDM5D, TBL1Y, BCORP1, PRY, DAZ,* and *AMELY*, which were classified as a mesoderm cluster. Endoderm markers were co-expressed with 7 MSY genes, i.e. *ZFY, TSPY, PRORY, VCY, EIF1AY, USP9Y*, and *RPKY*, which were grouped as an endoderm cluster. Finally, the ectoderm markers correlated with *TXLNGY, NLGN4Y, PCDH11Y, TMSB4Y, UTY, RBMY1,* and *HSFY* genes of the MSY, which were categorized as an ectoderm cluster. In contrast, 2 MSY genes, *SRY* and *TGIF2LY*, were more highly expressed in RH6 cells compared to EBs.

**Conclusion:**

We found a significant correlation between spontaneous differentiation and upregulation of specific MSY genes. The expression alterations of MSY genes implied the potential responsibility of their gene co-expression clusters for EB differentiation. We suggest that these genes may play important roles in early embryonic development.

## Introduction

Immediately after fertilization, the sex of the human embryo is determined by the spermatozoon carrying either a Y or an X chromosome (1). The sex chromosomes induce specific aspects of organ development in the absence of gonadal sex hormones (2). There are fundamental metabolic differences between female and male preimplantation embryos (3, 4). Briefly, three main aspects of sexual dimorphism have been observed including gene expression profiles, kinetics of growth, and embryonic mortality (5). Male embryos have a greater number of cells and metabolic activities than females with a significantly faster development (6-8).

Sexual dimorphisms are genetically initiated very early in embryonic development (9, 10); however the exact molecular mechanisms leading these differences remain to be comprehended. The sex chromosomes have conserved the essential sex-specific genes on a set of ancestral autosomes (11). Different chromosomal complements can display sexual dimorphism due to the different expression patterns of genes during preimplantation development (12, 13). The X chromosome is inactivated in the differentiated state of human embryonic stem cells (hESCs), causing the same content of the X chromosome in both sexes (14). Otherwise, it typically results in premature abortion and fetal death (15). The Y chromosome is the major genetic difference between sexes and plays an important role in male embryos especially at the preimplantation stage of early fetus development. The Y chromosome size is approximately 60 Mb containing two distinct segments. The male-specific region of the Y chromosome (MSY) contains genes specific to sexual dimorphism and undergoes no meiotic crossing over with a homolog. Two pseudo-autosomal regions flank the MSY on both sides and frequently undergo X−Y crossing over at male meiosis. (16). There are 47 genes on the MSY region as described in NeXtProt, of which 26 genes are validated at protein level (PE1), 11 genes at transcript level (PE2), 3 genes at homology base (PE3) and 7 genes at uncertain level (PE5) (www.nextprot.org, v2.23.1).

The Chromosome-Centric Human Proteome Project (C-HPP) has been established to identify all proteins encoded by each human chromosome (17, 18). The Y-Chromosome Human Proteome Project (Y-HPP), as part of C-HPP, identifies and annotates protein products of the Y chromosome genes using many methods including the cellbased approache, as one of the most important approaches (19). By taking advantage of hESCs, we can show how Y-HPP has been conducted to gain a rich understanding of the MSY genes during development. Two individual characteristics of hESCs make them well-matched for this kind of studies. First, hESCs provide a unique self-renewal capacity and an abundant source for proteomics analysis. Second, hESCs offer an interesting opportunity for simulating human embryonic development *in vitro* by generating all cell types related to the three germ layers (20). 

In hESCs, a range of tissue-specific differentiation is initiated via the formation of tissue-like spheroids called embryoid bodies (EBs) (21). EBs are 3-dimensional ESC aggregates that can determine the major genes involved in early embryogenesis following the lineage events to form three germ layers (mesoderm, endoderm, and ectoderm) (21-23). The lineage-specific differentiation of EBs *in vitro* recapitulates those seen in the developing embryo *in vivo* (24). On the other hand, EBs establish a model to simulate the *in vivo* differentiation process of ESCs under *in vitro* conditions to find the missing proteins by analyzing their expression levels and study possible effects of the human Y chromosome genes during spontaneous differentiation of hESCs (25-27). Although the expression profile of MSY genes has been transcriptionally detected in human pluripotent stem cells (28), a systematic expression profiling at the early developmental stages is needed. Here we aimed to determine a dynamic pattern of 38 MSY gene expressions at the early developmental stages of hESCs into EBs by analyzing transcriptional data.

## Materials and Methods

### Cell culture

This experimental study was carried out in accordance with the guide for the care and use of laboratory animals and approved by the Local Ethical Committee of Royan Institute for Stem Cell Biology and Technology with a code number IR.ACECR.ROYAN.REC.1396.15.

In this study, Royan H6 (RH6), a human embryonic stem cell line, was cultured on a mouse embryonic fibroblast (MEF) feeder layer. MEFs were mitotically inactivated prior to the addition of the RH6 cells by adding mitomycin C (10 µg/mL, Sigma, Netherlands). The base media for hESC was prepared with a combination of DMEM / F12 (Gibco) supplemented with 20% knockout serum replacement (KOSR, Gibco), 1% nonessential amino acids (Gibco), 1% insulin-transferrin-selenium (ITS, Invitrogen), 0.1mM beta-mercaptoethanol (Sigma, Germany), and 100 units/mL penicillin and 100μg / mL streptomycin (Gibco). Human recombinant bFGF (Basic fibroblast growth factor) (Royan Biotech, Iran) was added to the hESC media (final concentration, 12 ng/ml) at the seeding time. The cell cultures were incubated at 37˚C in a 5% CO_2_ atmosphere with daily media changes. The cells were passaged upon reaching 70% confluence. Then, RH6 cells were cultured on a thin Matrigel layer in hESC media containing 100 ng/ml bFGF free of any feeder cells for induction of an efficient differentiation. Freshly coated-Matrigel plates were prepared at least 2 hours prior to seeding the cells, according to manufacturer’s instructions. Briefly, for a 6-well plate, 500 μL of diluted Matrigel solution was used per well and incubated at 37˚C to be polymerized. RH6 cells were directly seeded on the wet Matrigel coated plate and allowed to settle for 30-90 minutes in an incubator (5% CO_2_, 37˚C) before flooding them with culture media. The hESC media was carefully added to each sample well. The cultures were maintained for 7 days, with daily media changes to form the RH6 colonies.

### Dynamic suspension of expanded RH6

After two passages on Matrigel, the RH6 cells were transferred to 125 mL spinner flask (Cellspin; Integra Biosciences AG, Switzerland) at a 40rpm agitation rate. For large-scale expansion, a 100-ml working volume was used as previously described (29). Briefly, undifferentiated RH6 cells were cultured with the optimal starting concentration of 2−3×10^5^ cells/mL at the hESC media, which was conditioned by MEFs, fresh 10 mM Rhoassociated kinase inhibitor (ROCKi; Sigma, Netherlands) and 100 ng/mL bFGF. The spinner flask was placed on a magnetic stir plate in an incubator at 37˚C and 5% CO_2_ without changing media during the first two days. RH6 cells were expanded in a 3D-dynamic suspension culture after 4 days. 

### Spontaneous differentiation of RH6 into EBs

**Fig.1:**
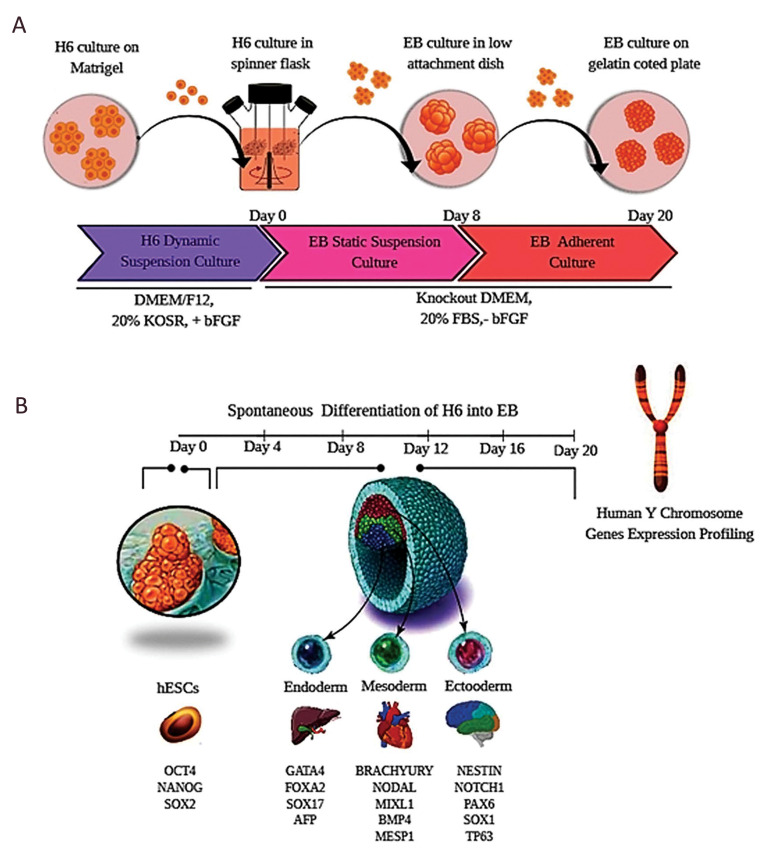
Schematic view of spontaneous differentiation of RH6 cell suspension into EBs. **A.** Dynamic suspension culture of RH6 in spinner flasks was followed by a two-stage differentiation into EBs in static suspension and adherent cultures, sequentially. **B.** Several time points were found for expression analysis of the pluripotent and differentiation markers. The expression of MSY genes was also investigated to compare with the markers in three germ layers of EBs. RH6; Royan H6, EBs; Embryoid bodies, and MSY; The male-specific region of the Y chromosome.

In the current study, RH6 cells were grown on inactivated feeder layers to gain the growth factors, cytokines and nutrients required for maintaining pluripotency. The cells were then transferred onto Matrigel (Sigma, Germany) to be free of any feeder cells and were prepared for a successful differentiation. The same size aggregates were generated from single cells in a dynamic suspension system and spontaneously differentiated into three embryonic germ layers of EBs. RH6 3D aggregates were formed in controlled sizes and shapes by optimizing the agitation speed, the impeller type and the incubation density for 4 days. The homogeneously sized colonies (175 ± 25 μm, approximately) were used to generate EBs by inducing spontaneous differentiation in static suspension condition for 20 days. The EB differentiation media consisted of KnockOut DMEM/F-12 base media, supplemented with 20% fetal bovine serum (FBS; Hyclone), 0.05 mM betamercaptoethanol, 1% glutamine (Gibco), 1% essential amino acids, 100 units/mL penicillin and 100 μg/mL streptomycin. For spontaneous differentiation, RH6 aggregates were cultured as a static suspension system in a 6-cm ultra-low attachment dish containing 5 ml of bFGF-free media for 8 days. The culture media was changed every 2 days. On day 8, RH6 aggregates were transferred into 0.1% gelatincoated plates to maintain spontaneous differentiation in a 2D cell culture system for 12 days, hence undergoing a 20-day differentiation. Samples were collected at several time points (0, 4, 8, 12, 16 and 20 days) for expression analysis of the pluripotency and differentiation markers in comparison to the MSY genes in early embryonic development (Table 1, See supplementary online information at www.celljournal.org). A schematic summarizing the different steps to generate EBs is shown in Figure 1.

### Ribonucleic Acid Isolation and Quantitative RealTime PCR (qRT-PCR)

According to the manufacturer’s protocol, total Ribonucleic acid (RNA) was isolated using TRIzol reagent (Invitrogen, USA). The purified RNA was reverse-transcribed into cDNA. Quantitative real-time PCR (qRT-PCR) was performed in the Rotor Gene 6000 (Corbett, Australia). *GAPDH* was used as the housekeeping gene. Finally, relative changes in gene expression levels were calculated by the threshold (quantification) cycle. The primer sequences (pluripotent and the three layer-specific markers) are shown in a Table 2, See supplementary online information at www.celljournal.org). The highly specific primers were designed for MSY genes using Vector NTI software (Life Sciences, USA) and verified with NCBI Primer-BLAST (https://www.ncbi.nlm.nih. gov/tools/primer-blast) (30). The primer sequences (MSY genes and transcript variants) are shown in a Table 3, See supplementary online information at www.celljournal.org.

### Statistical Analysis

Statistical analysis was performed for three biological replicates of each gene. Data are presented as mean ± SEM. Statistical significance was detected using a twoway ANOVA (^∗^ P<0.05) in Graphpad Prism software (Graphpad Software, USA). The relative expressions were compared to D0. Heatmap was generated using the heatmap.2 and g-plots libraries in the statistical software R (http://www.r-project.org). Heatmap was used to generate gene co-expression clusters based on pairwise Spearman correlations. Each square determined the correlation value between expression profiles of two genes. According to matched expression profiles, hierarchical clustering trees of the genes were shown in the top and left sides. The circo map was created with circos software (http://www. circos.ca). 

## Results

### Generation of the three embryonic germ layers 

**Fig.2:**
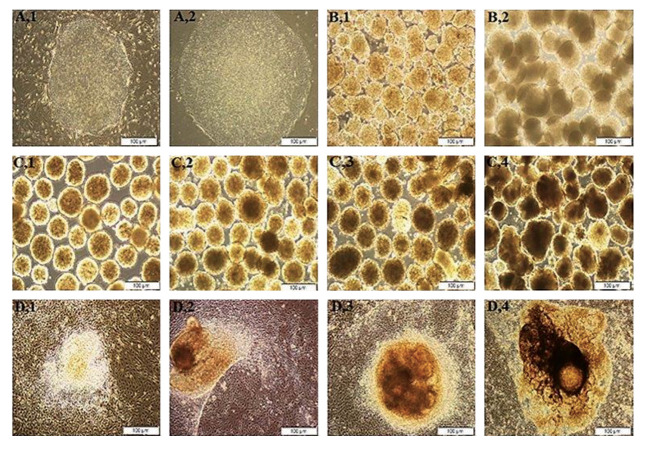
Morphology of RH6 colonies and EBs. **A.** Undifferentiated RH6 showed typical flat colonies with tight edge on the feeder layer (A1) and Matrigel (A2). **B. **The Single cells aggregated in a dynamic suspension culture for 4 days. On the first 3 days, RH6 created spheroid clumps or disc-like structures with a homogenous population of small cells (B1). On days 4, the aggregates were the same size (B2). **C.** In a static suspension culture, RH6 aggregates spontaneously differentiated into the EBs for 8 days. The aggregates of equal sizes on day 2 (C1) changed to the cystic and dense regions after 4 days (C2). EBs showed a dark cavity containing three germ layers on days 6 and 8, respectively (C3 and C4). **D.** 3D aggregates continued spontaneous differentiation in a 2D cell culture system for more than 12 days. Scale bar is 100µm. RH6; Royan H6, EBs; Embryoid bodies. , 3D; Three dimensional (3D) -dynamic suspension culture, and 2D; Two dimensional (2D) cell culture system.

To study the role of MSY genes in early embryonic development, RH6 cells were induced to differentiate spontaneously into the three embryonic germ layers of EBs. Stem cells were initially cultured on MEFs and Matrigel as a feeder layer and complex protein matrix, respectively, to maintain self-renewal and pluripotency (Fig .2A). Then, in a 3D dynamic suspension culture, RH6 single cells formed colonies with the same size and retained the characteristics of an undifferentiated hESC. Stem cell aggregates grew as a homogenous population of small cells forming spheroid clumps with distinct borders (Fig .2B). Differentiation was spontaneously induced through two sequential steps. At first, the aggregates with equal sizes made distinct cystic structures in a static suspension culture and closely compacted as a dark cavity in the center of the spheroid clumps like a solid ball. Therefore, EBs were well-organized with 3 germ layers which enlarged several times (Fig .2C). In the next step, EBs were cultured on a gelatin-coated plate as 2D culture systems to sequentially generate endodermal and ectodermal layers (Fig .2D).

### Expression of pluripotency and layer-specific markers during differentiation

We investigated the expression of some specific markers to evaluate cellular pluripotency and spontaneous differentiation at several time points (0, 4, 8, 12, 16 and 20 days). QRT-PCR was used to investigate the expressions of pluripotency markers (*OCT4, NANOG*, and *SOX2*), as well as some layer-specific markers including mesoderm (*NODAL, MIXL1, BMP4, MESP1*, and *BRACHYURY* (T)), endoderm (*GATA4, FOXA2, SOX17, and AFP*), and ectoderm (*NESTIN, NOTCH1, PAX6, TP63 and SOX1*) markers.

**Fig.3:**
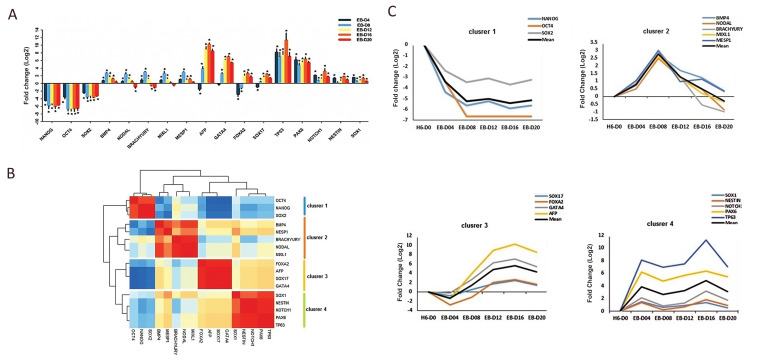
Analysis of expression patterns of pluripotent and layer-specific markers during EB differentiation. **A.** The expression pattern of pluripotent and three germ layer markers. **B.** Co-expressions of 17 markers in EB differentiation. **C.** Expression profiles for genes were shown in four clusters. Y-axis represents the difference between expression of the genes of a particular cluster and the mean expression of this cluster during time points suggesting the number of standard deviations that a particular data point differs from the mean. All data were presented as mean ± SEM (*P<0.05). EBs; Embryoid bodies.

For assessment of spontaneous differentiation, we also compared the expression of pluripotency and layer-specific markers in all samples. Although layerspecific markers showed very low expression levels in undifferentiated cells, they increased during RH6 differentiation (Fig .3A). Spearman correlation was applied by Heatmap to identify clusters with highly similar temporal expression patterns at several time points. Our analysis showed four distinct marker clusters (Fig .3B). The first cluster consisted of pluripotency markers including *OCT4, NANOG*, and *SOX2*, which are highly expressed in stem cells. The expression of pluripotency markers were significantly reduced or absent following the initiation of differentiation (Fig .3C). The second cluster consisted of 5 markers, including *NODAL, MIXL1, BMP4, MESP1*, and *BRACHYURY* (T), which were more expressed during spontaneous EB differentiation. It was indicated by an increased expression region for mesoderm markers on days 8, approximately, followed by a gradual decrease (Fig .3C). The third cluster consisted of 4 markers including *GATA4, FOXA2, SOX17*, and *AFP*, which are endoderm markers. Hierarchical clustering dendrogram of *GATA4, SOX17*, and *AFP* were more correlated than *FOXA2*. Endoderm markers showed a more identical pattern in comparison with the rest of the clusters (Fig .3C). The expression pattern of endoderm markers indicated a transient suppression until day 4 by differentiation initiation, following an enhancement on days 12-16 and ultimately a reduction on day 20 (Fig .3C). The fourth cluster including *NESTIN, NOTCH1, PAX6, TP63*, and *SOX1* were ectoderm markers. The expression pattern of *NESTIN, NOTCH1, PAX6*, and *TP63 *were more correlated compared to *SOX1*. They were moderately upregulated at the beginning of differentiation and downregulated on day 8. However, the expression of the ectoderm markers slowly increased from day 12 up to day 16, when the maximum expression level was observed. Our results showed an upregulation of the ectoderm markers on days 4 and 16. From day 16 to 20, however, their expressions slowly decreased (Fig .3C).

### The expression pattern of MSY genes in EB

The X-degenerate, X-transposed and ampliconic segments are euchromatic sequences of the MSY region of the Y chromosome [16]. The *NLGN4Y, SRY, ZFY, TXLNGY, AMELY, EIF1AY, GYG2P1, DDX3Y, UTY, RPS4Y1, USP9Y, TBL1Y, KDM5D, TMSB4Y, PRKY*, and *RPS4Y2* genes are located on the X-degenerate segment. The X-transposed sequences encode the *TGIF2LY* and *PCDH11Y* genes, and the ampliconic segment encodes 8 gene families namely *DAZ, CDY, VCY, HSFY, RBMY, TSPY, BPY2*, and *PRY* (31, 32). 

We investigated the expression pattern of Y chromosome genes to determine the genes involved in early EB differentiation. In general, our analysis was performed for 24 genes at protein evidence level (PE1), 8 genes at transcript evidence (PE2), 3 genes inferred from homology levels (PE3), as well as 3 genes at uncertain protein level (PE5), that have been demonstrated in Figure S1, See supplementary online information at www.celljournal.org).

**Fig.4:**
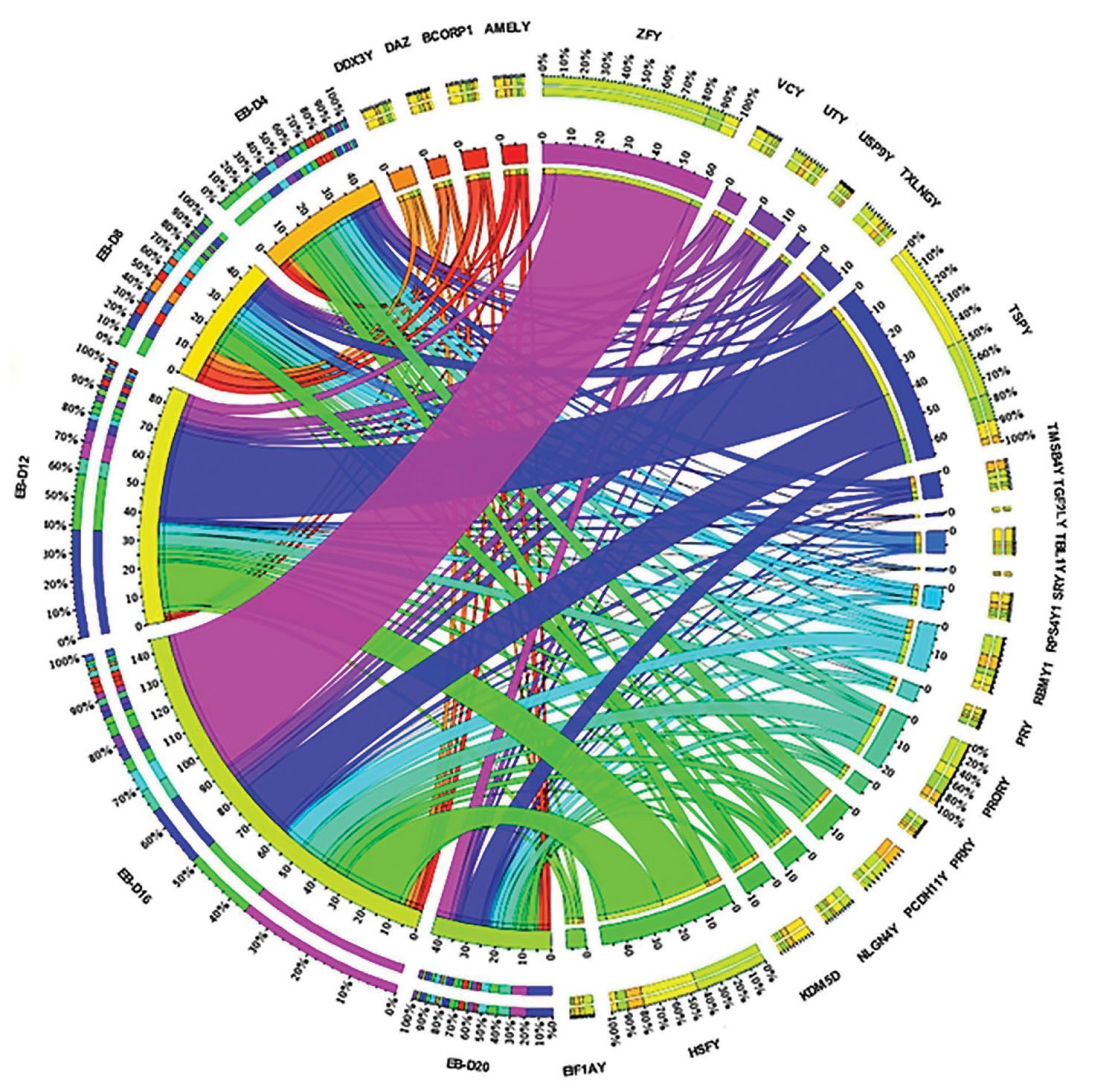
Schematic of dysregulated MSY transcripts during spontaneous differentiation. Circos map compared the expression pattern of 38 MSY genes at several time points (0, 4, 8, 12, 16 and 20 days). The expression of genes was related to time points of differentiation by inner arcs. The MSY genes were sorted in a descending order. Thus the *ZFY* gene, for example, had the maximal upregulation (0-60) at EB-D16 (~ 35%) while *SRY* and *TGIF2LY* indicated the maximal downregulation (0-2) during EB differentiation. MSY; The male-specific region of the Y chromosome and EBs; Embryoid bodies.

MSY genes showed altered expression levels during EB differentiation, as summarized in Figure 4. The expression pattern of 38 MSY genes was compared by Circos map at several time points (0, 4, 8, 12, 16 and 20 days, Fig .4). The inner colored segments were representative for each specific gene and the outer segments demonstrated the differentiation time points. The green segment, for example, was related to the *ZFY* transcript showing the maximum expression on days 16 (EB-D16, 85%), 12 (EB-D12, 5%) and 20 (EB-D20, 10%), respectively. The outside segment of differentiation time points represented the contribution of the Y chromosome genes in each group (i.e. in EBD16, *ZFY* had the highest fold change ~ 35% among MSY genes). The inner segment of differentiation time points showed that most of the MSY genes were upregulated at EB-D16 (0-145).

**Fig.5:**
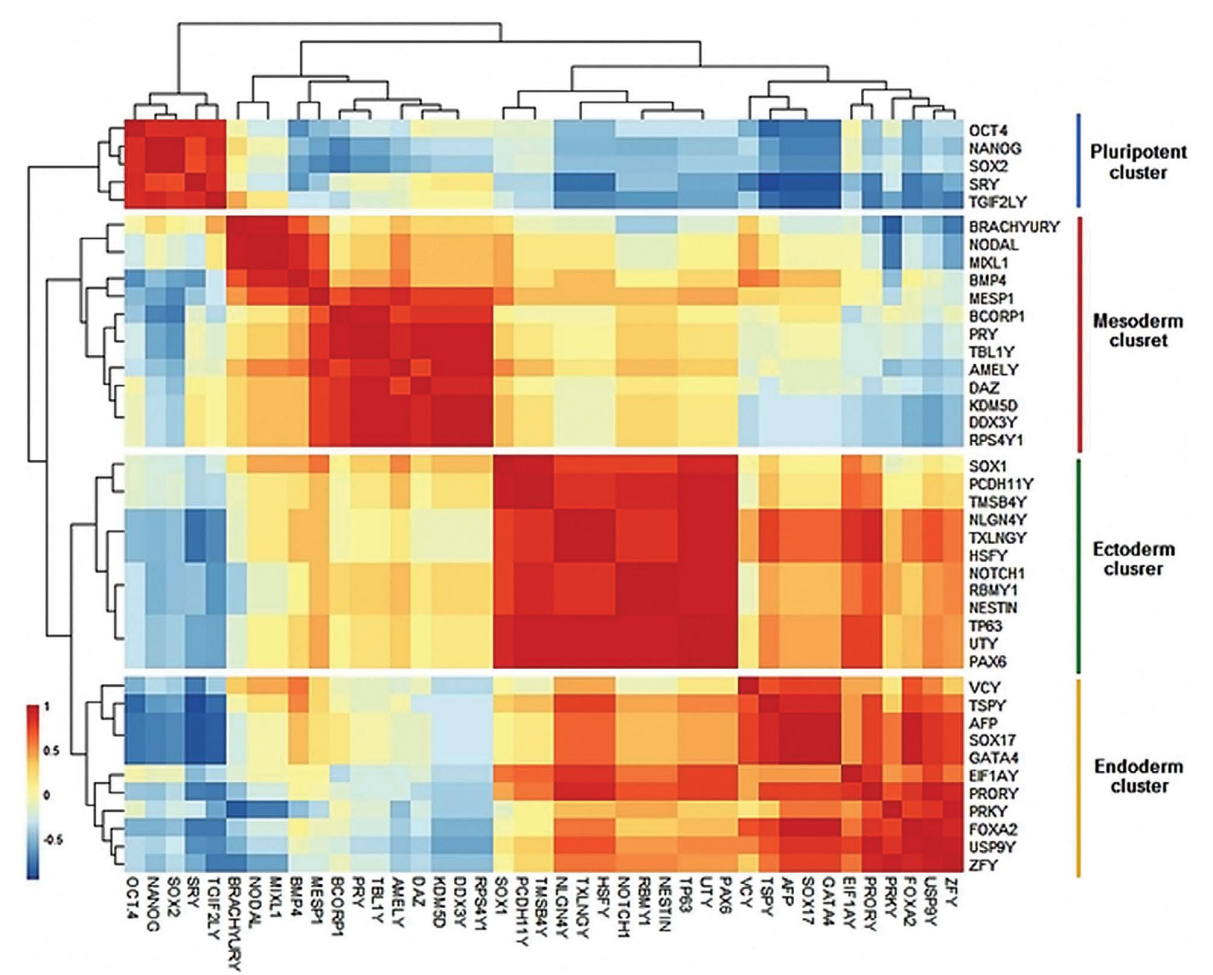
Co-expression of MSY genes and EB markers. The correlation of two expression profiles was determined by one square. Hierarchical trees were constructed based on matched profiles, shown on the top and left sides. Also, mesoderm, ectoderm, endoderm and pluripotent clusters were shown on the right side. MSY; The male-specific region of the Y chromosome and EBs; Embryoid bodies.

**Fig.6:**
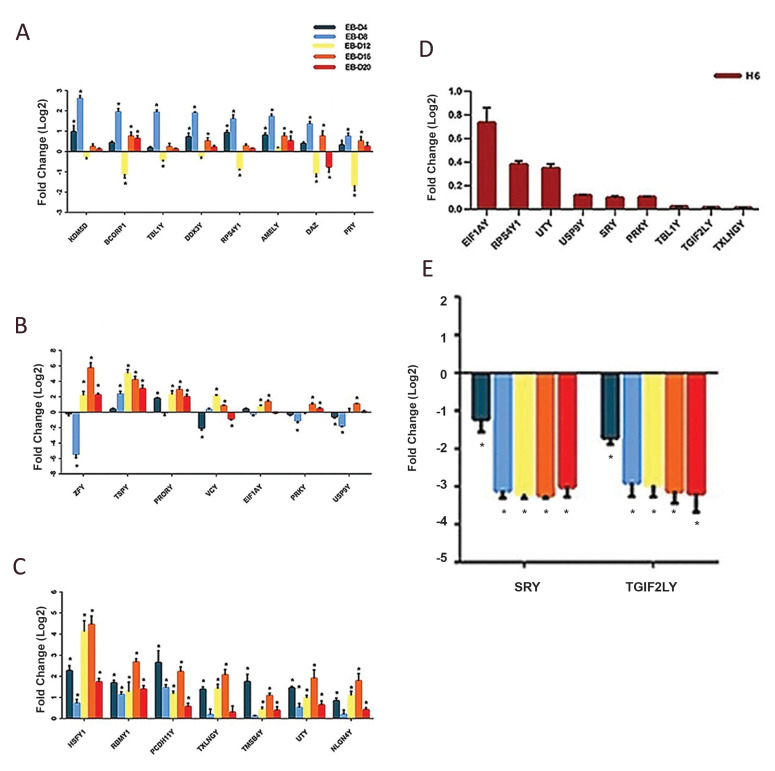
The expression pattern of MSY genes during spontaneous differentiation. **A. **The expression profile of MSY genes in the mesoderm cluster. **B** and **C.** The expression profile of MSY genes in the endoderm and ectoderm clusters, respectively. **D. **The expression profile of MSY genes in the pluripotency state in RH6 cells. **E.** The expression profile of MSY genes in the pluripotent cluster. All data were presented as mean ± SEM. *; P<0.05. MSY; The male-specific region of the Y chromosome and RH6; Royan H6.

The expression of MSY genes and EB markers were compared by Spearman’s Heatmap. The results detected four clusters, which contained highly correlated genes (Fig .5). The mesoderm markers showed high correlation with 8 MSY genes including *KDM5D, DDX3Y, RPS4Y1, TBL1Y, BCORP1, PRY, DAZ*, and *AMELY *(Fig .5). These genes were highly increased by differentiation initiation up to day 8 and were categorized as a mesoderm cluster (Fig .6A). The endoderm cluster was arranged based on a high correlation between endoderm markers and the 7 MSY genes: *ZFY, TSPY, PRORY, VCY, EIF1AY, USP9Y*, and *RPKY* (Fig .5). Among the mentioned genes, *VCY* and *TSPY* showed more correlation with endoderm markers by the Hierarchical clustering trees (Fig .6B). The ectoderm markers were grouped with 7 genes known as *TXLNGY, NLGN4Y, PCDH11Y, TMSB4Y, UTY, RBMY1*, and *HSFY* as an ectoderm cluster (Fig .5). The expression pattern of *PCDH11Y *and *TMSB4Y* demonstrated a high correlation with *SOX1*, which was increased by differentiation initiation on day 4 followed by a severe downregulation by day 16. In contrast, the expression pattern of *RBMY1, HSFY, TXLNGY, NLGN4Y*, and *UTY*, which were upregulated from day 12 to the end, was similar to *NESTIN, NOTCH1, PAX6*, and *TP63* as ectoderm markers (Fig .6C). Some of the MSY genes including *EIF1AY, PRKY, RPS4Y1, SRY, USP9Y, UTY, TXLNGY, TBL1Y*, and *TGIF2LY *showed higher expression levels in RH6 cells (Fig .6D). The Spearman’s analysis showed that pluripotency markers were more closely correlated with *SRY* and *TGIF2LY* genes, which were highly expressed in RH6 cells, but decreased by initiation of differentiation and remained at low levels throughout the differentiation period (Fig .6E). These genes were classified as a pluripotent cluster (Fig .5).

## Discussion

The Y-HPP was instituted to achieve a complete knowledge about the function, quantification, subcellular localization, and expression pattern of human Y chromosome protein and genes, especially during embryonic development. In the direction of one of the Y-HPP goals, we analyzed the expression pattern of most MSY genes in the process of male-ESCs (RH6) spontaneous differentiation into EBs as an *in vitro* model for early embryonic development. Our results indicated a higher expression of *SRY* and *TGIF2LY* genes in undifferentiated male embryonic stem cells compared to EBs. This observation is in agreement with previous studies showing the upregulation of *SRY* gene in fibroblasts reprogrammed to become induced pluripotent stem cells (iPSCs), and the downregulation of* SRY* following knockdown of both *OCT4* and *NANOG* genes (33, 34). Therefore, *SRY* promoter region has been shown to contain multiple putative *OCT4* and *NANOG* recognition sites. By starting of differentiation, we observed a significant reduction of *SRY* and *TGIF2LY*, following downregulation of *OCT4*, *NANOG* and *SOX2* markers corresponding to previous studies (28, 35).

Ronen and colleagues (2014) suggested that the MSY genes, including *RPS4Y1, DDX3Y, EIF1AY, TXLNGY, NLGN4Y, TMSB4Y, UTY, USP9Y, TTTY15, PRKY*, and *ZFY* were highly expressed in male-hESCs and iPSCs [33]. Jangravi and colleagues (2012) also demonstrated that hESC lines were enriched for *NLGN4Y, PRKY, PCDH11Y, TMSB4Y, USP9Y, RPS4Y2, TXLNGY, AMELY, UTY*, and *RPS4Y1* transcripts (28). Consistent with these studies, we showed that some of MSY genes including* EIF1AY, PRKY, RPS4Y1, SRY, USP9Y, UTY, TXLNGY, TBL1Y*, and *TGIF2LY* were specifically expressed in the pluripotent RH6 cells. 

Petropoulos and colleagues (2016) indicated that the expression of Y chromosome genes increased in male embryos since day 8, whereas the X chromosome genes were more expressed in female embryos on days 3 and 4. Therefore, X genes were gradually downregulated after 5 days in return for the upregulation of Y genes starting on day 8. Petropoulos’ study has shown that 10 of the Y chromosome genes, *DDX3Y, TXLNGY, TMSB4Y, PRKY, RPS4Y1, USP9Y, UTY, KDM5D, ZFY*, and *EIF1AY*, were upregulated at embryonic days and lineages, unlike *SRY* gene (35). Zhou and colleagues (2019) found that the Y chromosome is initially activated by *RPS4Y1* and *DDX3Y* genes in 8-day-old embryos when the two X chromosomes in females were widely activated in embryonic genome activation (36). 

Torres and colleagues (2013) showed that *Utx* was not required for the proliferation of knockout mouse embryonic stem cells (mESCs), but this gene contributed to the establishment of ectoderm and mesoderm. In *Utx* knockout ESCs, *Uty* as a homologue of *Utx* could compensate for some of the functions of *Utx* during ectoderm and mesoderm differentiation (37). Vakilian and colleagues (2015) successfully differentiated a human embryonic carcinoma cell line (NTERA-2) into neuronal cells. They showed that the expression of 12 MSY genes, specifically *EIF1AY, RBMY1, DDX3Y, HSFY, BPY2, UTY, PCDH11Y, USP9Y, RPS4Y1, SRY, ZFY *and *PRY* were significantly upregulated during neural differentiation (38). Meyfour and colleagues (2017) performed a cardiac differentiation and reported an upregulation for the* TBL1Y, KDM5D, PCDH11Y, USP9Y, ZFY, RPS4Y, DDX3Y, XKRY, PRY*, and *UTY* genes at early mesoderm differentiation, and the* BCORP1, RBMY* and *HSFY* genes during late cardiogenesis. On the other hand, the expression of 5 MSY genes namely *VCY, SRY, TXLNGY, NLGN4Y* and *TMSB4Y* were decreased at early differentiation stages (39). Tsugata and colleagues )2015( performed differentiation of both male and female PSCs into insulinproducing cells and demonstrated that *RPS4Y1, DDX3Y, EIF1AY*, and *NLGN4Y* genes were expressed at higher levels in a male cell line compared to the female cells (40). In the current study, we found a significant correlation between spontaneous differentiation and upregulation of most MSY genes. Briefly, it appears that *KDM5D, TBL1Y, RPS4Y1, DDX3Y, PRY, DAZ, BCORP1*, and *AMELY* genes play key roles in mesoderm layer development. In the same manner, *TXLNGY, NLGN4Y, PCDH11Y, TMSB4Y, UTY, RBMY1*, and *HSFY* genes contribute to establishment of the ectoderm layer. Also, *ZFY, TSPY, PRORY, VCY, EIF1AY, USP9Y*, and *RPKY* genes were involved in the endoderm differentiation. 

## Conclusion

The present study is the first report to genetically investigate MSY genes during spontaneous differentiation of RH6 into EBs. Using Spearman’s Heatmap we identified distinct gene co-expression clusters to validate the correlation of MSY genes with each germ layer. The expression alterations characterized the potential responsibilities of each cluster for the differentiation of mesoderm, ectoderm and endoderm layers. We suggest that these genes may play important roles in early embryonic developments of males. Our results, along with future studies on directed differentiations, are potentially essential for a better understanding of gender-specific factors in embryonic developmental differences.

## Supplementary PDF


