# Supplementary material for: The Contribution of Y Chromosome Genes to Spontaneous Differentiation of Human Embryonic Stem Cells into Embryoid Bodies In Vitro
Source: Cell J. 2021 Mar 1;23(1):40–50. doi: 10.22074/cellj.2021.7145 (PMC7944136; doi:10.22074/cellj.2021.7145)
Supplement: Supplementary file 1 [file Cell-J-23-40-s01.pdf]

www.celljournal.org

Supplementary Information for

The Contribution of Y Chromosome Genes to Spontaneous Differentiation of Human Embryonic Stem Cells into Embryoid Bodies *In Vitro*

Simin Nafian Dehkordi, M.Sc.[1, 2,] Farzaneh Khani, M.Sc.[1, 2], Seyedeh Nafiseh Hassani, Ph.D.[3], Hossein Baharvand, Ph.D.[3], [4],

Hamid Reza Soleimanpour-lichaei, Ph.D.[1*], Ghasem Hosseini Salekdeh, Ph.D.[2, 5, 6*]

1. Department of Stem Cells and Regenerative Medicine, Institute of Medical Biotechnology, National Institute of Genetic Engineering and Biotechnology (NIGEB), Tehran, Iran
2. Department of Molecular Systems Biology, Cell Science Research Center, Royan Institute for Stem Cell Biology and Technology, ACECR, Tehran, Iran
3. Department of Stem Cells and Developmental Biology, Cell Science Research Center, Royan Institute for Stem Cell Biology and Technology, ACECR, Tehran, Iran
4. Department of Developmental Biology, University of Science and Culture, Tehran, Iran
5. Department of Systems Biology, Agricultural Biotechnology Research Institute of Iran, Karaj, Iran
6. Department of Molecular Sciences, Macquarie University, Sydney, NSW, Australia

**Corresponding Addresses: P.O.Box: 14965-161, Department of Stem Cells and Regenerative Medicine, Institute of Medical Biotechnology, National Institute of Genetic Engineering & Biotechnology (NIGEB), Tehran, Iran.*
P.O.Box: 16635-148, Department of Molecular Systems Biology, Cell Science Research Center, Royan Institute for Stem Cell Biology and Technology, ACECR, Tehran, Iran
Emails: hrs@nigeb.ac.ir, hsalekdeh@yahoo.com

Table S1:Y chromosome genes, their status in neXtProt and findings on their roles in development

Sequence class	Gene symbol	Gene name	Function	Human organogenesis	Protein existence	Copy numbers
X –transposed	*TGIF2LY*	TGFB-Induced Factor 2-Like, Y-Linked	DNA binding, protein binding	-	PE1	1
	PCDH11Y	Proto cadherin 11 Y-linked	Calcium ion binding	Brain, Heart	PE1	1
X-degenerate	*SRY*	Sex-determining region Y	Transcription factor, DNA binding	Brain, Testis, kidney	PE1	1
	RPS4Y1	Ribosomal Protein S4, Y-Linked 1	RNA binding, rRNA binding	Brain, Heart	PE1	1
	ZFY	Zinc Finger Protein, Y-Linked	transcriptional activator	Brain, Heart	PE1	1
	AMELY	Amelogenin, Y-Linked	Structural constituent of tooth enamel	Tooth enamel development	PE2	1
	TBL1Y	Transducin β Like 1, Y-Linked	Transcription activation/co-repression	Brain, Heart	PE1	1
	PRKY	Protein Kinase, Y-Linked	Protein serine/ threonine kinase	Brain, kidney	PE5	1
	USP9Y	Ubiquitin Specific Peptidase 9, Y-Linked	ubiquitin-specific protease, Hydrolase	Brain, Heart, Spermatogenesis	PE1	1
	DDX3Y	DEAD-Box Helicase 3, Y-Linked	RNA helicase, DNA binding, RNA binding	Brain, Heart, Spermatogenesis	PE1	1
	UTY (KDM6C)	Ubiquitously Transcribed TPR, Y-Linked	histone demethylase	Brain, Heart	PE1	1
	TMSB4Y (TYB4Y)	Thymosin β 4, Y-Linked	Actin monomer binding	Brain	PE1	1
	NLGN4Y	Neuroligin 4, Y-Linked	Cell adhesion molecule binding	Brain	PE2	1
	BCORP1	BCL6 Corepressor Pseudogene 1, Y-Linked	unknown function	heart	PE5	1
	TXLNGY (CYorf15A)	Taxilin γ Pseudogene, Y-Linked	Syntaxin binding	Brain	PE5	1
	KDM5D (SMCY)	Lysine-specific Demethylase 5D	Histone demethylase, DNA binding	Brain, Heart	PE1	1
	EIF1AY	Eukaryotic Translation Initiation Factor 1A, Y-Linked	Translational initiation, Protein binding	Brain	PE1	1

Table S1: Continuded

Sequence class	Gene symbol	Gene name	Function	Human organogenesis	Protein existence	Copy numbers
Amplicon	*TSPY*	Testis-specific protein, Y-Linked	Protein binding	Spermatogenesis	PE1,PE2,PE3	6of 32
	VCY (BPY1)	Variable Charge, Y-Linked	unknown function	-	PE1	2
	HSFY	Heat Shock Transcription Factor, Y-Linked 1	Transcription factor, , protein binding	Brain, Heart	PE1	2
	PRORY (CYorf17)	Proline Rich, Y-Linked	unknown function	-	PE2	1
	RBMY	RNA binding motif protein, Y-Linked	RNA splicing, Protein binding	Spermatogenesis, Heart	PE1,PE2	6
	PRY	PTPN13-like, Y-Linked	unknown function	Spermatogenesis, Heart	PE1	2 of 4
	DAZ	Deleted In Azoospermia	RNA binding, translation activator	Spermatogenesis	PE1	4

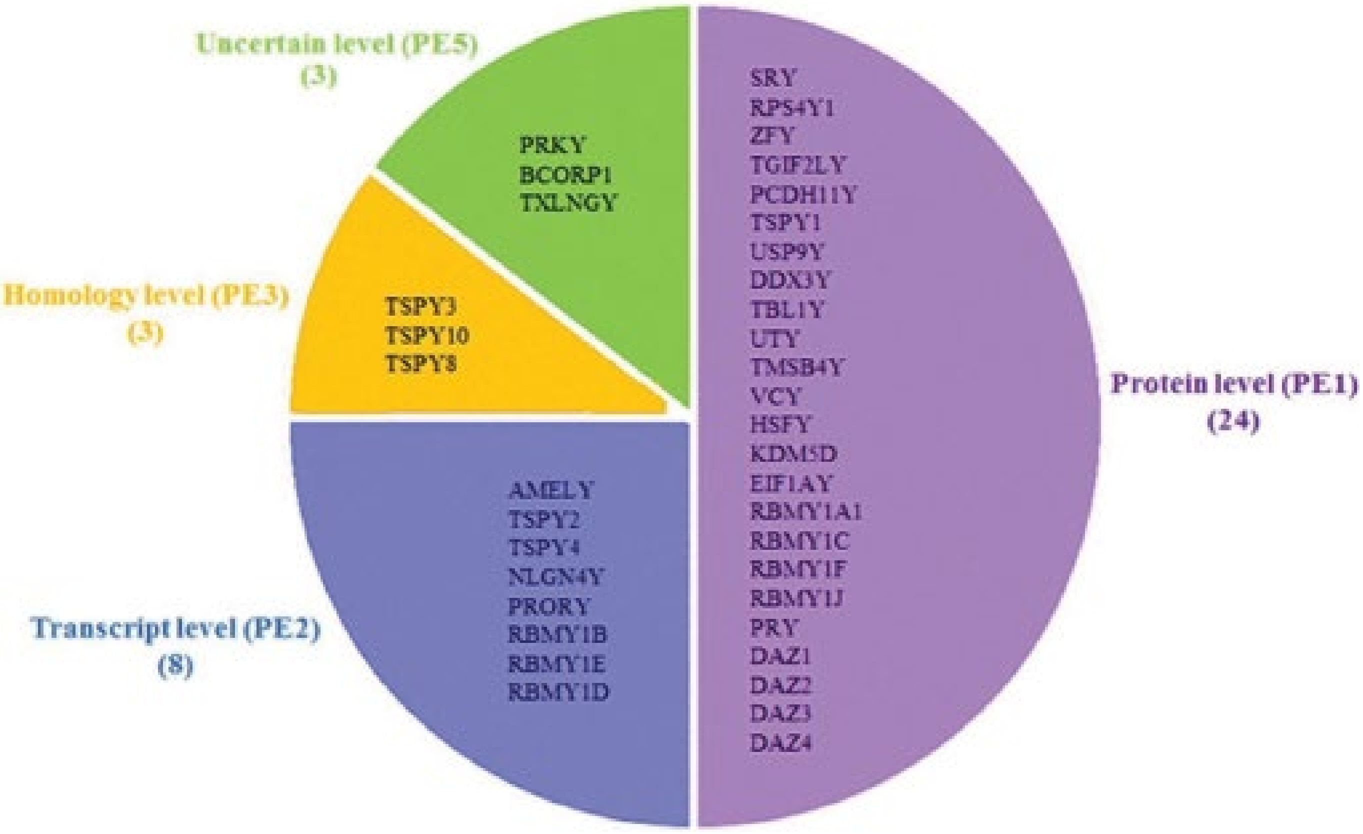

Fig.S1: NeXtProt classification of MSY genes which were analyzed in this study. (www.nextprot.org, v2.22.8).

Table S2: List of specific primers designed for pluripotent and three layer-specific markers using Gene Runner software

Gene Symbol	Forward sequence (F)<br>Reverse sequence (R)	Target genes and transcripts
GAPDH	F: 5´-GAAATCCCATCACCATCTTCC-3´<br>R: 5´-GGCTGTTGTCATACTTCTCAT-3´	*GAPDH*<br>(Transcript variant 1-4)
OCT4	F: 5´-CTGGGTGATCCTCGGACCT-3´<br>R: 5´-CACAGAACTCATACGGCGGG-3´	*OCT4*<br>(*POU5F1*)
NANOG	F: 5´-AAAGTCTTAAAGCTGCCTTAAC-3´<br>R: 5´-CAGTCGGATGCTTCAAAG-3´	*NANOG*<br>(Transcript variant 1,2)
SOX2	F: 5´-GGGAAATGGAAGGGGTGCAAAAGAGG-3´<br>R: 5´-TTGCGTGAGTGTGGATGGGATTGGTG-3´	*SOX*2
SOX1	F: 5´-CACAACTCGGAGATCAGCAA-3´<br>R: 5´-GGTACTTGTAATCCGGGTGC-3´	*SOX1*
NESTIN	F: 5´-TCCAGGAACGGAAAATCAAG-3´<br>R: 5´-GCCTCCTCATCCCCTACTTC-3´	*NESTIN*
NOTCH1	F: 5´-CAGACCCACACCCAGTA-3´<br>R: 5´-GGCAACGTCAACACCTT-3´	*NOTCH1*
TP63	F: 5´-TTTCAGAGGCAATCCACACA-3´<br>R: 5´-ATGCATGCAAATGAGCTCTG-3´	TP63<br>(P63)
PAX6	F: 5´-GTCCATCTTTGCTTGGGAAA-3´<br>R: 5´-TAGCCAGGTTGCGAAGAACT-3´	*PAX6*
AFP	F: 5´-AAATGCGTTTCTCGTTGCTT-3´<br>R: 5´-GCCACAGGCCAATAGTTTGT-3´	*AFP*
GATA4	F: 5´-CCT GTC ATC TCA CTA CGG-3´<br>R: 5´-GCT GTT CCA AGA GTC CTG-3´	*GATA4*
FOXA2	F: 5´-GGAGCGGTGAAGATGGAA-3´<br>R: 5´-TACGTGTTCATGCCGTTCAT-3´	*FOXA2*
SOX17	F: 5´-CTCTGCCTCCTCCACGAA-3´<br>R: 5´-CAGAATCCAGACCTGCACAA-3´	*SOX17*
MIXL1	F: 5´-TCTTGAGGTAGATGTGAACTG-3´<br>R: 5´-CTTTGAACCAATGTCTTCAGAG-3´	*MIXL1*
BRACHYURY	F: 5´-AATTGGTCCAGCCTTGGAAT-3´<br>R: 5´-CGTTGCTCACAGACCACA-3´	*BRACHYURY*<br>(T)
NODAL	F: 5´-GCGTACATGCTGAGCCTCTA-3´<br>R: 5´-GGTGACCTGGGACAAAGTG-3´	*NODAL*
MESP1	F: 5´-ACCTTCGAAGTGGTTCCTTG-3´<br>R: 5´-TCCTGCTTGCCTCAAAGTGT-3´	*MESP1*
BMP4	F: 5´-GGCCAGCATGTCAGGATTAG-3´<br>R: 5´-CACATCGCTGAAGTCCACAT-3´	*BMP4*

Table S3: List of specific primers designed for the Y chromosome genes usingVector NTI software

Gene Symbol	Forward sequence(F)<br>Reverse sequence(R)	Target genes and transcripts
AMELY	F: 5´-GAGGACCAAGCCTCCCTGTGTAGCA-3´<br>R: 5´-ATAACCAGGGTGCCCAGGATGAGG-3´	*AMELY*
BCORP1	F: 5´-TTTGTCTTAGTCAACTGTCCCA-3´<br>R: 5´-GACCTTGCCTCATAGCCA-3´	*BCORP1*<br>(Transcript variant 1&2)
DAZ	F: 5´-TGTTCCAGCGGACTTCACCAGC-3´<br>R: 5´-TTGCAGCAGACATGGTGGTGGC-3´	*DAZ1, DAZ2, DAZ3, DAZ4*
DDX3Y	F: 5´-AACCCTGTCAAGTCTGTCGAGCCTC-3´<br>R: 5´-GCGATCCACGGTGGTTTGAATACA-3´	*DDX3Y* (SMCY)<br>(Transcript variant 1)
EIF1AY	F: 5´-GGAAGAGGTCTCACGAGGCTGTCAT-3´<br>R: 5´-GCTTCCAATCGTCCATTTCCCA-3´	*EIF1AY*<br>(Transcript variant 1,2)
HSFY	F: 5´-TTCATGGGATGAGAATGGAAC-3´<br>R: 5´-GAAAGGTGGCTAGAAAGGCAG-3´	HSFY1,HSFY2<br>(Transcript variant 1, 2, 3)
KDM5D	F: 5´-AGCAGAGCATTTGGAGGAGG-3´<br>R: 5´-TCCCCTGCACACTGGTTTGT-3´	*KDM5D*<br>(Transcript variant1, 2, 3)
NLGN4Y	F: 5´-GCCAGCTATGGGAACGTCATCG-3´<br>R: 5´-CTCCCACACAAACGTGCCTCTTGC-3´	*NLGN4Y*<br>(Transcript variant 1)
PCDH11Y	F: 5´-CAAACTGTCACAAGTGTTTG-3´<br>R: 5´-CTGCATAGTAGTTGTCAAGG-3´	*PCDH11Y*<br>(Transcript variant a, b, c, d)
PRKY	F: 5´-CATAAACTGAGGGTGTGGGGTCTGG-3´<br>R: 5´-GTCTACAGCCAAGTTTCCAGCCAGG-3´	*PRKY*
PRORY	F: 5´-CCTCCTGCCTCTCCATAT-3´<br>R: 5´-TCATCCATGACCACAGACG-3´	PRORY (CYorf17*)*
PRY	F: 5´-CAACCTTCTTTCACTGACACCCACC-3´<br>R: 5´-TTGTCCTTGAGTGGTCTCTTGGG-3´	*PRY, PRY2*
RBMY1	F: 5´-TGCCACATAACTTGAGCTACC-3´<br>R: 5´-ACTCGAGCTGTGGTGATTTTC-3´	*RBMY1A1, RBMY1B, RBMY1D,*<br>*RBMY1J, RBMY1F, RBMY1E*
RPS4Y1	F: 5´-AGGAAGATTACTGTGGGAGTG-3´<br>R: 5´-GTATCGTTCACCTTGATGAC-3´	*RPS4Y1*
SRY	F: 5´-CGAAACTCAGAGATCAGCAAGCAGC-3´<br>R: 5´-CCTGTAATTTCTGTGCCTCCTGG-3´	*SRY*
TBL1Y	F: 5´-CCTCAGTGTGGGGAATGCCAGC-3´<br>R: 5´-CCAGCAGGCTTCTCAGCATCCA-3´	*TBL1Y*<br>(Transcript variant 2)
TGIF2LY	F: 5´-GAAACAACAGTAACGATAAGCCTC-3´<br>R: 5´-TGCCTGTATCTGCGTTATTTCTCG-3´	*TGIF2LY*
TMSB4Y	F: 5´-TGTTTCTCACGCTCACTTTGGATTG-3´<br>R: 5´-TGCCTGTTTAAGATTCGCCTGC-3´	*TMSB4Y (TYB4Y)*

Table S3: Continuded

**Gene Symbol**	**Forward sequence(F)**<br>**Reverse sequence(R)**	**Target genes and transcripts**
TSPY	F: 5´-CATCACAGAATACAGGGCTTCTC-3´<br>R: 5´-TAGATCCTGCGAAGTTGTGGT-3´	*TSPY1,TSPY2, TSPY3,*<br>*TSPY4,TSPY8, TSPY10*
TXLNGY	F: 5´-TTCCTGAGCAAGAAGTAGCC-3´<br>R: 5´-CTTTCCTCCAGAAGATCAGCA-3´	*TXLNGY* (CYorf15A)<br>(Transcript variant 2)
USP9Y	F: 5´-GACCCTTGTGTATCAGCAGCATTTC-3´<br>R: 5´-GCTGCCCAGTAATGACTAAGTCCA-3´	USP9Y
UTY	F: 5´-TGCTGCAACAAGAGCTTCTC-3´<br>R: 5´-TGGCACGAAATATCAAAGTCTC-3´	*UTY (KDM6C)*<br>(Transcript variant 1-77)
VCY	F: 5´-CGCCCATCTACTCCCCTATCTCCCT-3´<br>R: 5´-ACATGGGAAGCACCCTGCTGGTGA-3´	*VCY, VCY1B*<br>*(BPY1)*
ZFY	F: 5´-ATTTCATTTTGTTCAGCAGAC-3´<br>R: 5´-TCCAAAATTCAGTGAGCAG-3´	*ZFY*<br>(Transcript variant 1, 2, 3)
